# Imaginative play for inclusion: evaluating the young dragons tabletop role-playing intervention in UK schools

**DOI:** 10.1186/s12889-026-28225-z

**Published:** 2026-06-20

**Authors:** Austen El-Osta, Yiran Li, Sami Altalib, Aos Alaa, Rebecca Linsley, Sarah Newman, Etiene Steyn, Garry Harper, David Coulter, Cornelia Junghans Minton

**Affiliations:** 1 Self-Care Academic Research Unit (SCARU), Imperial College London School of Public Health, London, W12 0BZ, UK; 2 School of Life course & Population Sciences King’s College London, London, WC2R 2LS, UK; 3 Department of Primary Care & Public Health, School of Public Health, Imperial College London, 90 Wood Lane, London, W12 0BZ UK; 4 Royal Borough of Kensington and Chelsea, Westminster City Council, 64 Victoria St, London, SW1E 6QP, UK; 5 Mythic Minds CIC, Carter Court, Southall, London, UB2 4GU, UK

**Keywords:** Tabletop role-playing games (TTRPG), Dungeons & Dragons, Social and emotional learning (SEL), School exclusion, Emotional regulation, Inclusion, Adolescent mental health, Mixed-methods evaluation, Realist evaluation, Self-management, Teamwork, Narrative-based intervention, At-risk pupils, UK schools

## Abstract

**Background:**

School exclusion in England disproportionately affects pupils with social, emotional, and mental health needs. Tabletop roleplaying games (TTRPGs) may strengthen social emotional learning but are rarely evaluated in UK state schools. Young Dragons, a Dungeons & Dragons based programme, was developed to support emotional regulation, teamwork, and engagement among pupils at risk of exclusion in two London boroughs.

**Methods:**

A convergent mixed methods realist evaluation was delivered across ten schools. Pupils aged 9–16 attended weekly one-hour sessions for 6–8 weeks in small, consistent groups. Pre/post pupil surveys captured wellbeing, self-concept, peer relations, school belonging, and loneliness; matched pair change (*n* = 22) used Wilcoxon signed rank tests. Semi-structured interviews with school staff and facilitators and observation of multiagency meetings explored implementation and perceived impact. Findings were integrated using joint displays and context–mechanism–outcome (CMO) mapping.

**Results:**

No statistically significant within person change was detected across matched items (all *p* > 0.10). Distributions showed heterogeneous trajectories: many pupils reported better mood, anger regulation, and confidence, while a minority shifted toward greater disengagement or loneliness. Exploratory analysis of routine school records (*n* = 30) showed a significant reduction in mean suspensions from 0.7 pre-intervention to 0.0 post-intervention (*p* = 0.022) and a modest, non-significant increase in mean attendance from 90.6% to 92.7% (*p* = 0.069). Qualitative accounts described strong engagement, psychological safety, and visible gains in self-management, turn taking, and teamwork, with positive spillover into classroom behaviour when groups were stable and facilitation was consistent. Delivery challenges included timetable pressures, space constraints, and stigma around targeted provision. Integration identified skilled facilitation, small-group safety, and structured reflection as key mechanisms enabling co-regulation, perspective taking, and belonging.

**Conclusion:**

Young Dragons was feasible and acceptable in high need school settings. Benefits were mechanism-consistent for many participants but contingent on context and facilitator quality. These pilot data justify a larger, controlled evaluation to test causal pathways and longer-term outcomes for inclusion and emotional wellbeing.

**Supplementary Information:**

The online version contains supplementary material available at https://doi.org/10.1186/s12889-026-28225-z.

## Introduction

School exclusion is a persistent educational and public health concern in England with consequences that extend across the life course [1, 2]. Pupils who are suspended or permanently excluded are more likely to experience poor mental health, low attainment and later contact with the criminal justice system [2–4]. Recent national figures indicate record levels of suspensions and permanent exclusions, with local patterns that are unequally distributed and concentrated among children already facing adversity, including those with special educational needs and disabilities and those living in poverty [5, 6]. In Westminster and the Royal Borough of Kensington & Chelsea (RBKC), exclusion rates have at times exceeded London averages and are concentrated in the most deprived wards [6, 7]. Schools in these boroughs also contend with high proportions of pupils eligible for free school meals, substantial linguistic diversity and rising demand for mental health support [8–12]. The public health and equity implications are clear: exclusion is not simply a disciplinary endpoint, but a marker of unmet social, emotional and mental health (SEMH) need and local education systems require preventative, inclusive responses that can be delivered within the everyday ecology of schools [1].

Against this backdrop, there is growing interest in creative, low stigma, play based interventions that can strengthen social-emotional learning (SEL) while engaging pupils who may be ambivalent toward conventional support [13–15]. Tabletop roleplaying games (TTRPGs), notably Dungeons & Dragons (D&D), have emerged internationally as promising tools for cultivating communication, collaboration, perspective taking and problem-solving [14]. Yet their application in UK state schools remains rare, the evidence base is thin in formal education settings and, crucially, little is known about how such programmes are experienced and implemented in real classrooms serving diverse, high need cohorts.

The theoretical appeal of TTRPGs for SEL lies in the way structured narrative, rules and collaboration combine to create psychologically safe conditions for social risk taking and identity work [16, 17]. In D&D, pupils adopt characters, co-create stories, negotiate choices and contend with consequences inside a rule governed, facilitator led environment [16–18]. These mechanics align with the five competencies in the Collaborative for Academic, Social, and Emotional Learning (CASEL) framework self-awareness, self-management, social awareness, relationship skills and responsible decision making by requiring turn taking, empathic perspective taking, conflict navigation and reflective decision making in a shared enterprise [19, 20]. The format also supports coregulation: pupils practise sharing the spotlight, reading group norms and making joint plans, while the rules scaffold fairness and predictability [17, 18]. In short, the game world functions as a “safe laboratory” where pupils can rehearse different versions of themselves and transfer emerging skills into everyday school life [16].

Although therapeutic and community studies have reported improvements in anxiety, self-esteem, social connection and teamwork associated with TTRPG participation, the translation of these gains into mainstream UK schools and the conditions that enable or constrain them remain underexamined [15, 17, 19, 21, 22]. Teachers’ perspectives are especially underrepresented despite teachers’ pivotal role in observing day-to-day behaviours, judging acceptability and brokering practical fit with timetables, staffing and safeguarding routines [19]. Recent qualitative work embedded in central London schools suggests teachers observe changes across all five CASEL domains when TTRPG programmes are delivered in small, supported groups, but also identifies salient implementation questions: who benefits, under what conditions and how can delivery be sustained at scale without diluting quality.

The Young Dragons pilot was designed to address these questions in real school contexts. Commissioned by the BI Borough Children’s Services and delivered with Mythic Minds CIC across ten state schools in Westminster (WCC) and RBKC, the programme targeted pupils aged 9–16 identified by school staff as at risk of exclusion, socially isolated, or experiencing SEMH difficulties. Delivery consisted of weekly one-hour sessions over six to eight weeks in small, consistent groups of four to six pupils, facilitated by trained “storytellers” with school staff present in a supervisory or co-facilitative capacity. The core pedagogical logic combined imaginative play, cooperative problem solving and structured reflection, with the intention of building trust, communication, self-regulation and a sense of belonging. In practice, delivery had to accommodate the variability that typifies urban schooling: timetable pressures, room availability, attendance fluctuations, group composition and the need to adapt scenarios to the developmental stage and diversity of each cohort.

The primary aim of this realist evaluation is to assess the impact of the Young Dragons programme on the emotional wellbeing, social communication and school engagement of pupils aged 9–16 identified as at risk of exclusion or experiencing SEMH needs. Specifically, the evaluation sought to: (i) explore changes in self-esteem, emotional regulation and perceived school belonging because of programme participation, (ii) gather pupil-reported experiences of loneliness and inclusion before and after the intervention, (iii) identify delivery challenges and contextual factors influencing programme fidelity, engagement and acceptability and (iv) generate recommendations for refinement, scalability and future implementation across similar school settings.

## Methods

### Study design

This was a convergent mixed methods evaluation with a realist orientation, conducted as pre-post pilot in mainstream, through secondary and alternative provision schools in WCC and RBKC. The design was pragmatic, chosen to test feasibility and acceptability under real delivery constraints, to describe within person change in key social emotional domains and to develop explanatory accounts of how context and mechanisms conditioned outcomes. The mixed methods frame enabled parallel quantitative and qualitative analyses followed by structured integration to build context–mechanism–outcome (CMO) propositions.

### Setting, participants and recruitment

The intervention ran across ten state funded schools spanning primary, secondary, and all through academy and a pupil referral unit. School inclusion reflected the local mix of high deprivation, elevated free school meal eligibility, substantial proportions of pupils using English as an additional language and rising presentations of social, emotional and mental health need. Pupils aged 9–16 were nominated by special educational needs coordinators, heads of year, or inclusion leads based on observed risk of exclusion, SEMH challenges, social isolation, or disengagement. Exclusion criteria were minimal and limited to acute risk or placement in intensive specialist provision. Groups were kept small (typically 4–6) and pupils remained with the same peers to support cohesion, trust and narrative continuity. Pupils were informed that participation was voluntary and could decline or withdraw at any time without consequence, irrespective of parental or school consent. School staff were present during sessions in a supervisory or facilitative role.

### Intervention

Young Dragons comprised weekly one-hour sessions of the tabletop roleplaying game Dungeons & Dragons for six to eight weeks, delivered on school premises by trained facilitators from the social enterprise Mythic Minds CIC. Sessions followed a consistent arc of check-in and recap, facilitated narrative play, cooperative problem-solving and guided debrief with explicit links to emotions, group process and transfer to school life.

Sessions differed from typical recreational Dungeons & Dragons play in several respects. Narratives were purposefully structured to foreground cooperative problem-solving, emotional reflection and inclusive participation rather than competitive or open-ended gameplay. Facilitators actively scaffolded turn-taking, prompted reflection on emotional responses and group dynamics and adapted scenarios in real time to align with pupils’ developmental needs and behavioural regulation capacity. Debrief components explicitly linked in-game experiences to real-world school contexts, distinguishing the intervention from purely recreational formats.

The format leveraged structured rules, turn taking, character perspective taking and collective decision-making to exercise competencies aligned with the CASEL framework (self-awareness, self-management, social awareness, relationship skills, responsible decision making). Scaffolding and scenario complexity were adjusted to age, literacy and group dynamics. Fidelity was supported by facilitator supervision and weekly debriefs, while adaptation to timetable and space constraints preserved ecological fit.

### Quantitative measures and data collection

The survey was designed for feasibility and accessibility rather than psychometric validation; items were single-indicator measures aligned with key domains but may not fully capture construct breadth or demonstrate established reliability. Free-text items captured brief reflections on pupils’ experiences, including what they enjoyed and found challenging. Participants were asked to reflect both on overall group dynamics and, where appropriate, on individual pupil trajectories.

Pupil reported outcomes were collected immediately before the first gaming session and after the final session using a bespoke, age-appropriate survey codeveloped with educators and the delivery partner. Items captured emotional wellbeing, self-concept and confidence, emotional regulation (stress, anger control, feeling control), social communication and peer support, loneliness (direct measure) and school engagement (belonging and enjoyment). Response formats used simplified Likert scales and emoji aided anchors to enhance accessibility. Free text questions captured brief reflections. To preserve anonymity while enabling linkage, pupils used pseudonymous “hero names”; manual reconciliation of minor spelling variants was undertaken to maximise match accuracy. Surveys were administered in class time as paper or online forms with adult support when required. Data quality screening excluded test entries, nonconsent and irreconcilable records. The survey was disseminated on the Qualtrics XLM platform. The electronic survey is included in Supplementary File 1.

### Quantitative analysis

Descriptive statistics using frequencies and percentages summarised response distributions at each timepoint across domains for all available cases. Pre–post within person change for matched pairs was estimated using the Wilcoxon signed rank test for ordinal outcomes, reporting p-values and signed Z statistics. Statistical significance was set at 0.05. Data was analysed using STATA version 18 (StataCorp LP, College Station, TX, USA).

### Qualitative design, sampling and data collection

The qualitative strand adopted an interpretivist approach to capture the experiences of those closest to delivery, focusing on teacher perspectives on pupil change, implementation enablers and barriers and prospects for sustainability. Semi structured interviews were conducted with nine participants comprising school-based staff and facilitators; interviews lasted approximately 20–45 min, were held via Microsoft Teams and followed a topic guide covering general impressions, perceived impact across SEL domains, practicalities of delivery and future relevance. In parallel, nonparticipant observation of three multiagency steering group meetings provided contextual insight into coordination, adaptive strategies and process learning across sites. With consent, interviews were audio recorded for verbatim transcription; identifiable details were pseudonymised. Field notes from observations were recorded systematically; to reduce reactivity, direct session observation was avoided when it inhibited pupil spontaneity, with the team privileging post session debriefs for richer, less intrusive insight.

### Qualitative analysis

Transcripts were imported into NVivo 14 and analysed using thematic analysis following Braun and Clarke’s six phase procedure. Coding was inductive and data driven in the first cycle to preserve participants’ language and local meaning, with iterative development of candidate themes capturing pupil level changes, group dynamics, facilitation and organisational conditions. In a second analytic pass, themes were mapped onto the CASEL framework to identify which competencies were most salient and to test alignment between reported changes and the programme’s theory of change. Observational notes were not formally coded but were used for triangulation and to refine theme boundaries and interpretations. Analytic memos recorded decisions, reflexive considerations and deviant cases. COREQ informed documentation of researcher roles, data saturation rationale and procedures to enhance trustworthiness.

### Mixed methods integration and realist synthesis

Integration used a convergent design in which quantitative and qualitative strands were analysed independently and then brought together through joint displays and realist pattern matching. Joint displays aligned pre-post distributions and matched pair change patterns by domain with illustrative teacher quotations and facilitation narratives, enabling inspection of convergence, complementarity, or divergence. A realist heuristic guided the development of CMO propositions. For example, small, stable groups led by skilled facilitators (context) appeared to trigger coregulated play, perspective taking and collaborative problem solving (mechanisms), which for pupils with high baseline dysregulation manifested as improved anger control, teamwork and confidence (outcomes); conversely, timetable pressure, space changes, or unfamiliar adults (context) appeared to blunt engagement and attenuate gains in school belonging (outcome). Integration outputs were used to refine practical guidance and to specify testable hypotheses for future controlled or stepped wedge evaluations.

### Reporting standards and methodological frameworks

This study was designed and reported in accordance with established reporting and methodological guidance appropriate to each component of the evaluation. The quantitative observational component followed the Strengthening the Reporting of Observational Studies in Epidemiology (STROBE) guidelines. The qualitative interview component was reported in line with the Consolidated Criteria for Reporting Qualitative Research (COREQ) checklist. Integration of quantitative and qualitative findings followed the Good Reporting of A Mixed Methods Study (GRAMMS) framework.

Given the programme’s theory-driven, explanatory focus, a realist orientation was adopted to explore how outcomes varied by context. Development and reporting of context–mechanism–outcome configurations were informed by the Realist And Meta-narrative Evidence Syntheses: Evolving Standards (RAMESES II) guidance. Description of the Young Dragons intervention and its delivery was structured using the Template for Intervention Description and Replication (TIDieR) checklist to support transparency and replicability. Reporting followed the GRAMMS for mixed methods, STROBE (supplementary files 2) for the quantitative observational component, and COREQ (supplementary files 3) for the qualitative interviews.

## Results

### Participants and data completeness

Across ten schools, 86 pupil surveys were collected between May-July 2025. After data quality checks (removal of test entries, non-consent and irreconcilable identifiers), 50 baseline and 36 post-intervention surveys were retained. Of these, 22 pupils could be matched pre-post using pseudonymous “hero names”. The reduced matched sample reflects a combination of attrition between timepoints and inconsistencies in pseudonym recording (e.g., spelling variation or non-use), which limited deterministic linkage despite manual reconciliation procedures. Interview data comprised nine semi-structured interviews with school staff and facilitators, alongside non‑participant observations from three multi‑agency steering group meetings. No adverse events were reported. The Main survey findings are illustrated in Supplementary Table 1.

### Changes in Students’ Wellbeing and Self-Perception

Distributions at the group level were broadly stable but showed visible polarisation on several items. For overall mood (“How is everything going?”), the most positive category remained high (46.0% at baseline versus 50.0% post), while the most negative category emerged at follow-up (0.0% to 13.9%). School engagement showed a similar bifurcation:" "was 32.0% at baseline and 33.3% post, but"" increased from 2.0% to 16.7%. Self-concept showed mixed movement: endorsement of “I have a number of good qualities” increased in the “agree/strongly agree” range (66.0% to 69.5%) and “unsure” declined (22.0% to 13.9%), yet “I feel good about myself” saw “strongly disagree” rise (6.0% to 16.7%). For emotional regulation, anger control shifted positively (“true” 14.0% to 27.8%, “very not true” 24.0% to 11.1%), whereas stress coping remained challenging, with high “unsure” at both timepoints. Loneliness “often/always” rose from 2.0% to 8.3%. School belonging remained mostly moderate to high, but “not at all true” increased (12.2% to 19.4%); Supplementary Table 1.

### Quantitative outcomes

**Fig. 1:**
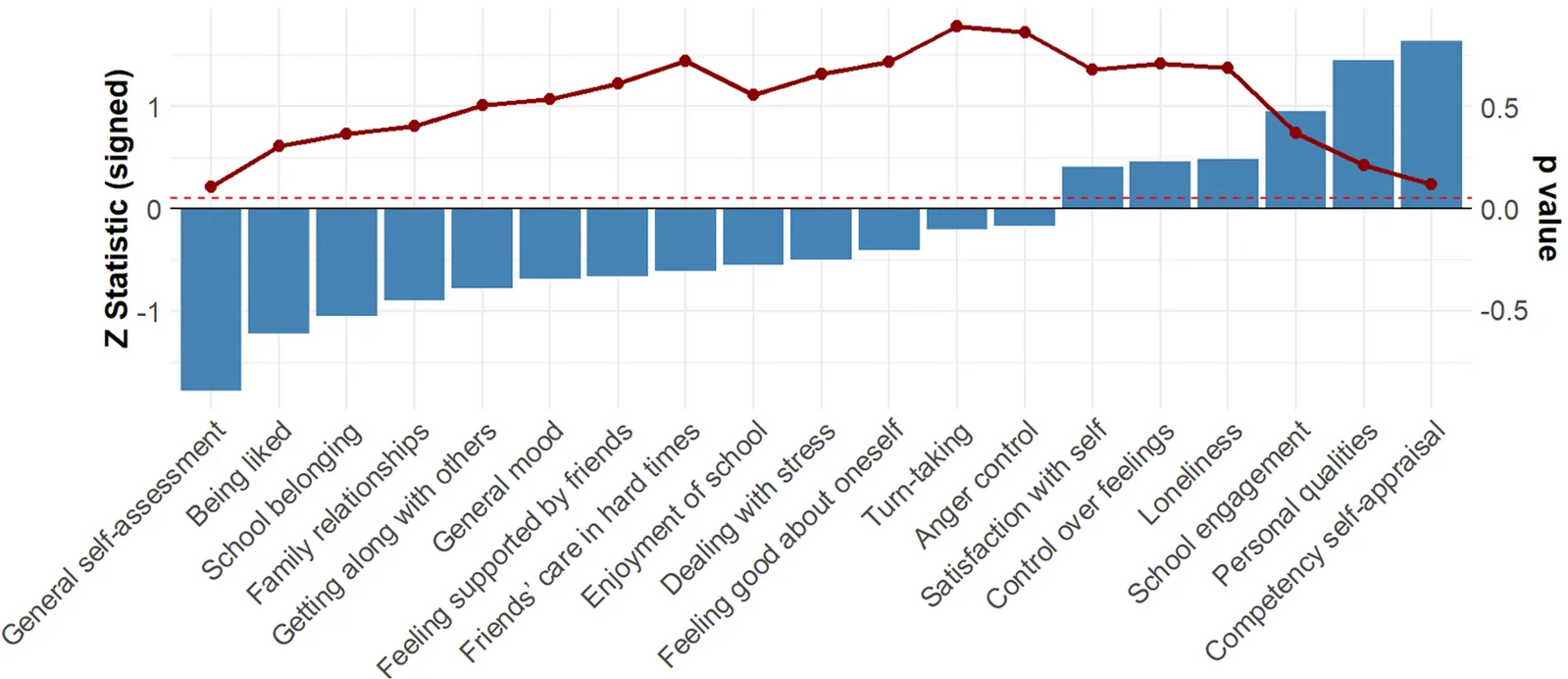
A combined bar and line chart with dual y-axes presenting Wilcoxon signed-rank test results across domains. Bars show signed Z statistics (direction and magnitude). Line and points indicate scaled p-values (significance). The red dashed line marks the *p* = 0.05 threshold

It should be noted that survey items varied in their scoring direction: for positively framed items, higher scores indicate better outcomes, while for negatively framed items, higher scores indicate greater difficulty. Accordingly, the sign of the Z statistic should be interpreted relative to each item’s framing rather than as a uniform indicator of improvement or deterioration. Matched-pair analysis of 22 pupils showed no statistically significant changes across any primary outcome items (Table 1; Fig. 1). However, directionally, several domains suggested small improvements after the intervention. General self-assessment (“How are you doing?”) showed the largest directional shift (Z = − 1.777, *p* = 0.107), noting that the sign reflects item coding rather than direction of improvement for post-intervention ratings. Measures of competence (“I am able to do things as well as most other people”) and personal qualities also trended upward (Z = 1.635 and 1.448, respectively). Indicators related to mood, school belonging, and social relationships remained broadly positive and stable from baseline to follow-up. Family relationships continued to score highly, with no deterioration observed. Measures of emotional control and stress coping showed small, non-significant fluctuations, suggesting overall stability rather than decline. While statistical significance was not achieved, descriptive trends indicated modest improvements in self-perceived competence, self-assessment, and peer support following the intervention. The pattern is consistent with feasibility-focused inference and heterogeneous trajectories rather than uniform group-level change.

**Table 1:** Matched pair Wilcoxon signed rank tests for primary outcome items (*n* = 22). All *p* > 0.10; negative Z indicates higher post scores on items framed positively and vice versa as reported in the source table

Domain / Item	Z	*p*
General mood “How is everything going?”	−0.684	0.541
School engagement “How are you doing at school?”	0.950	0.371
Family relationships “How are things in your family?”	−0.898	0.406
General self-assessment “How are you doing?”	−1.777	0.107
Satisfaction with self “I am satisfied with myself”	0.404	0.684
Personal qualities “I have a number of good qualities”	1.448	0.213
Competence “I am able to do things as well as most other people”	1.635	0.118
Feeling good about oneself “I feel good about myself”	−0.409	0.722
Control over feelings “I find it hard to control my feelings”	0.456	0.714
Stress-coping “I’m able to deal with stress”	−0.503	0.664
Anger control “I can control my anger when I want to”	−0.168	0.871
Getting along “I get along with people around me”	−0.779	0.508
Being liked “People like to spend time with me”	−1.219	0.307
Friend support “I feel supported by my friends”	−0.663	0.617
Friends’ care “My friends care about me when times are hard”	−0.610	0.727
Turn-taking “I take turns when playing games with others”	−0.207	0.899
Loneliness “How often do you feel lonely?”	0.480	0.694
School belonging “I feel like I belong at my school”	−1.051	0.366
Enjoyment of school “I enjoy coming to school”	−0.551	0.563

### Responder heterogeneity

Aggregated counts show concurrent improvements and deteriorations within the same cohort, highlighting nonuniform response (Table 2). For example, for overall mood, 6 pupils improved, 12 were unchanged and 4 worsened; for school engagement 5 improved and 8 worsened; for competence self-appraisal 11 improved and 5 worsened; for anger control 7 improved and 8 worsened. For items where “higher” reflects more difficulty (“hard to control my feelings”; loneliness), worsened counts capture movement toward greater difficulty. These distributions support a three-way typology suggested by the descriptive data: pupils who showed patterns consistent with improvement in several domains, pupils who remained broadly stable and pupils who shifted toward more negative responses postintervention. This structure is not visible in central tendency but is evident in category movement and individual trajectories.

**Table 2:**
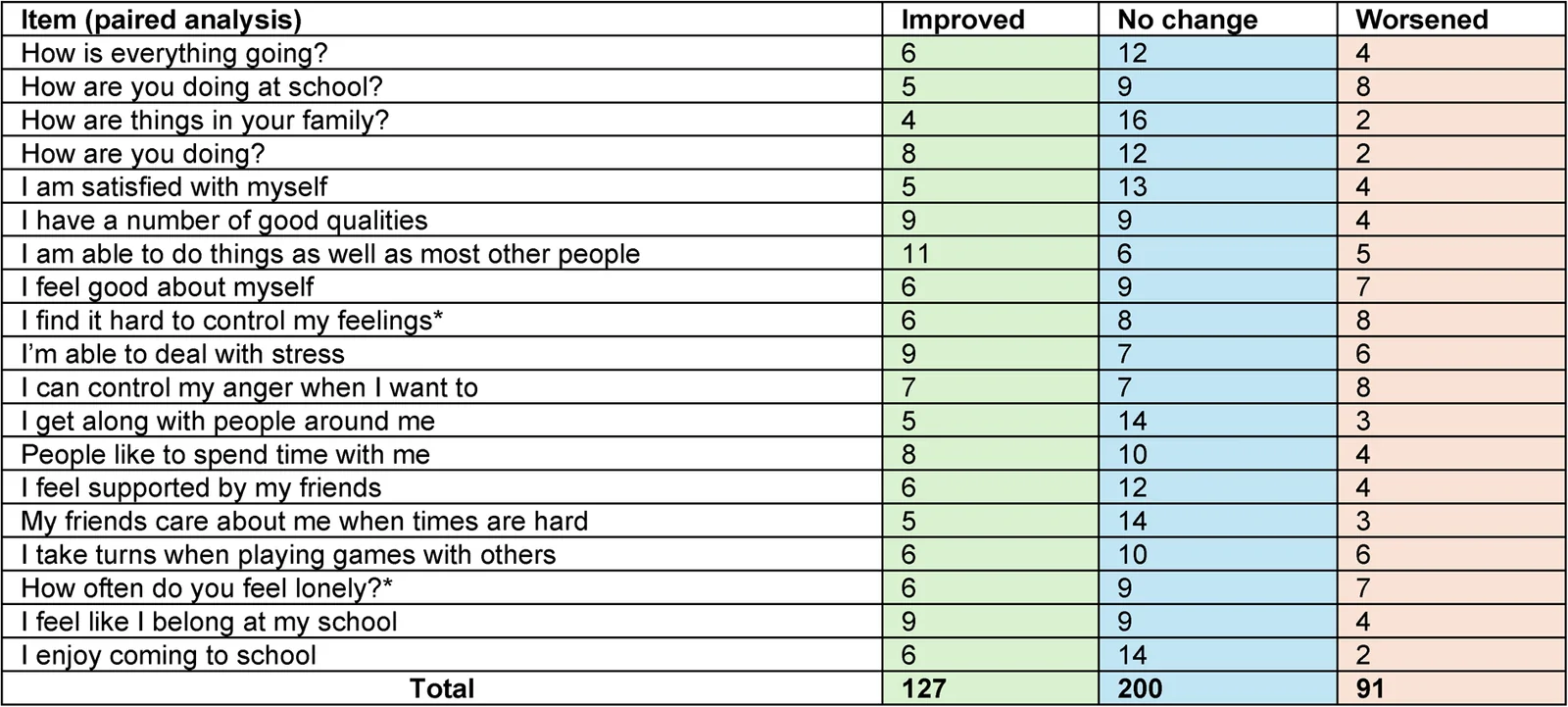
Paired response change counts by item (*n*=22)

*Higher post values on these items indicate greater difficulty (worse outcome)

**Fig. 2:**
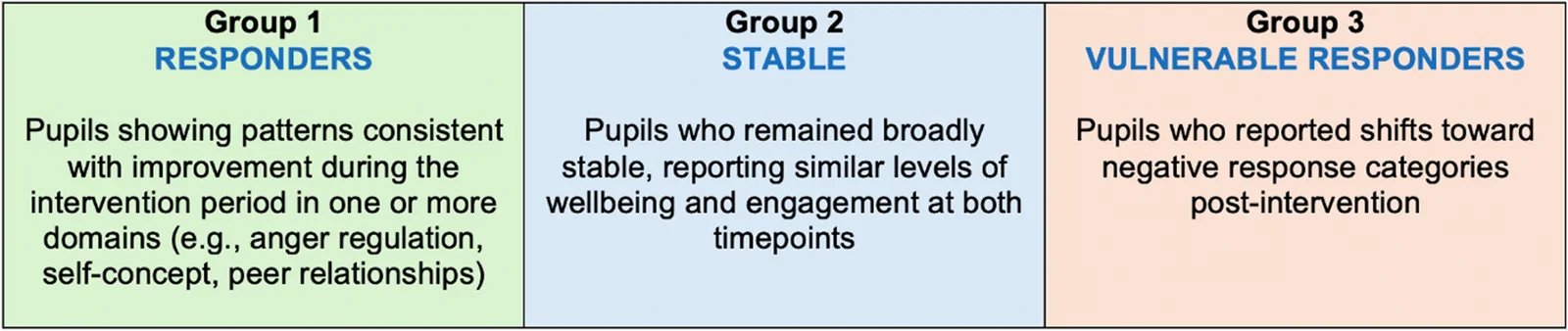
Impact typology

**Fig. 3:**
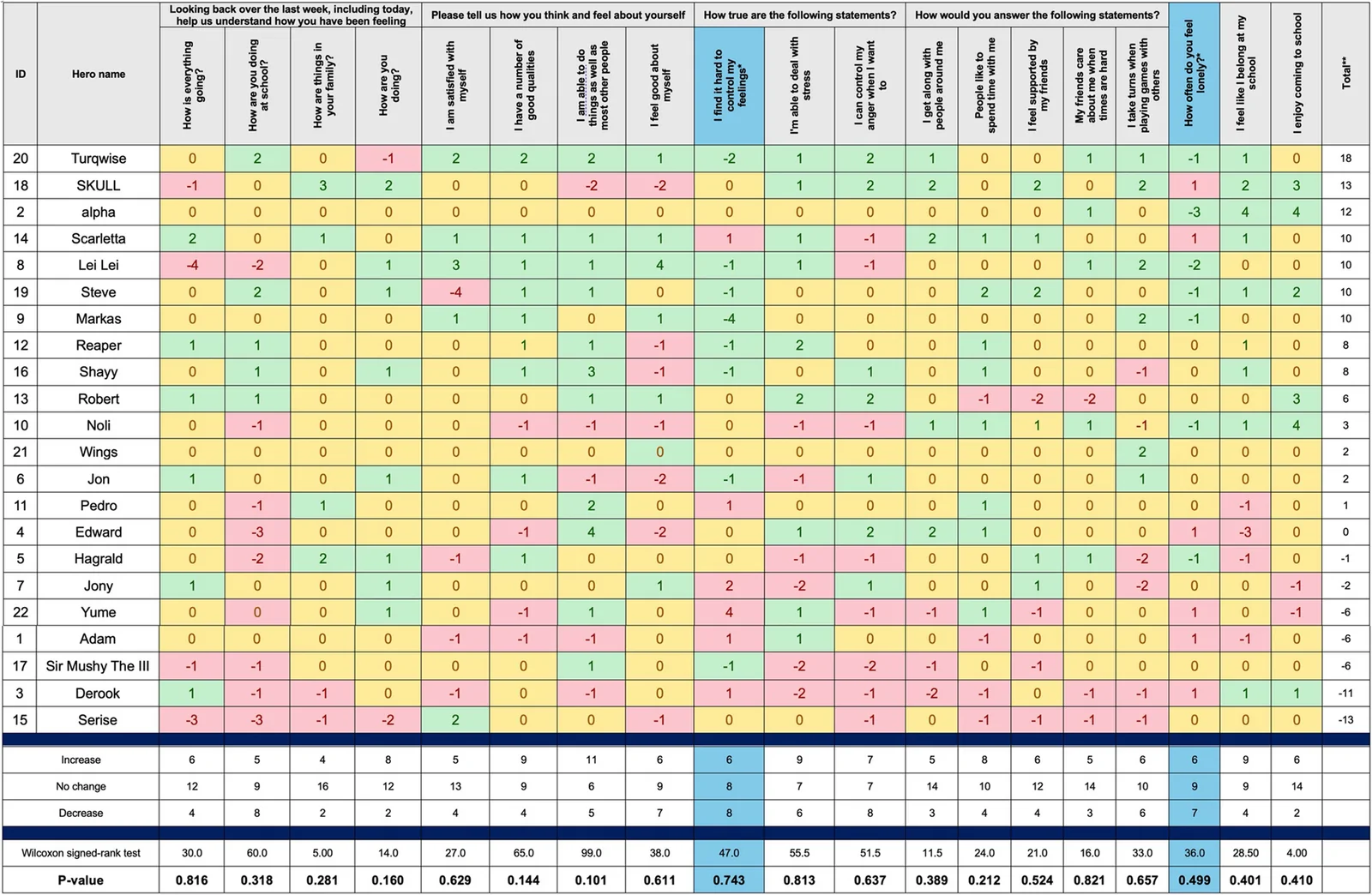
Coloured matrix of paired participant responses (*n* = 22). *For questions highlighted in blue, the colour coding was intentionally inverted: positive changes were shown in red, and negative changes in green, reflecting the specific nature and interpretation of these items. **In calculating the total score, for questions with inverted colour coding (highlighted in blue), the sign of the response was inverted (i.e., positive became negative and vice versa) before summing to ensure consistent interpretation across all questions.

Key demographic data from the 10 participating schools is shown in Table 3 below. Across 30 matched pupils with attendance and suspension data, mean attendance increased from 90.6% to 92.7%, although this did not reach statistical significance (*p* = 0.069). Importantly, mean suspensions declined significantly, from 0.7 pre-intervention to 0.0 post-intervention (*p* = 0.022). From the data, a three-way typology of impact can be inferred (Fig. 2). The presence of all three groups is a hallmark of early-stage, creative mental health programmes delivered in real-world, high-need school contexts. A confusion matrix (Fig. 3) provides a visual synthesis of paired participant feedback across key domains of the Young Dragons intervention. The matrix provides a visual synthesis of paired participant feedback across key domains of the Young Dragons intervention. Colours denote the nature of responses (positive, neutral, or negative), enabling at-a-glance comparison across participants and domains. Green denotes a Positive response (clear evidence of improvement or benefit), Amber denotes a neutral/mixed response (limited change or varied perspectives), Red denotes a negative response (no improvement or reported concern). 

**Table 3:** Participating school demographic data

	School Name	% FSM Eligible	% SEND Support	% SEN Education, Health & Care Plan (EHCP)	% Pupils 1st language not EN (2023/24)	% Suspension Rate (2023/24)
RBKC	St Francis of Assisi RC primary school	47.8%	15.6%	6.1%	39.7%	0.0%
	Servite RC primary school	20.5%	17.0%	5.5%	54.6%	0.0%
	Avondale Park primary school	49.5%	23.4%	5.5%	66.6%	8.8%
	Holland Park School	30.5%	13.6%	4.1%	36.3%	10.2%
	Kensington Aldridge Academy	54.3%	13.6%	7.1%	44.1%	22.1%
WCC	Gateway Primary	51.7%	13.0%	4.6%	89.8%	0.7%
	Queen’s Park Primary	47.7%	8.6%	10.5%	70.3%	1.1%
	King Solomon Academy	64.2%	18.6%	2.3%	66.0%	15.2%
	Westminster Academy	48.3%	15.0%	2.7%	56.2%	27.1%
	St Marylebone CofE School	22.0%	10.0%	3.0%	29.0%	2.5%

### Qualitative outcomes

This study explored the perceptions of educators, programme staff and a parent involved in the D&D pilot. A total of 7 interviews were conducted between 11 and 21 July. Participant characteristics are shown in Table 4.

**Table 4:** Participant characteristics

Participant role	School	Other notes	*N*.
Commissioners & Programme Leads	NA	Included commissioners, public health, programme managers	6
Mythic Minds CiC Intervention leads	NA	Deep overview of delivery of intervention	2
Pastoral support worker	Westminster Academy	Substituting for Y7 colleague, direct observations + staff feedback	1
Learning Support Assistant (LSA)	St Marylebone High School	Attended all but one session, participant-observer	1
Parent of pupil / School admin staff	Avondale Park Primary	Parent of pupil with SEND; also worked in school office	1
School learning mentor (teacher)	Avondale Park Primary	Accompanied children in the games sessions, witnessed increase in turn-taking, better collaboration as the imitative progressed	1
Headteacher	St Francis of Assisi Primary School	Did not attend sessions but spoke to children before and afterwards and engaged with the storyteller after the sessions	1
Headteacher	Queen’s Park Primary	Attended sessions and observed increase in confidence and turn taking. Being able to advocate for themselves.	1
Co-Head teacher with responsibility for children with additional needs	Gateway Academy (primary)	Accompanied children, witnessed improvement in turn taking, improved social interaction	1

Five overarching categories emerged, capturing socio-emotional gains, confidence and self-expression, social connection, creativity and implementation factors. These qualitative findings help contextualise the quantitative results, highlighting how the programme influenced pupils and the conditions that supported or constrained its delivery. Several themes emerged from the interviews which could be grouped into five categories (Table 5).

**Table 5:** Summary of qualitative themes from Young Dragons evaluation

Category	Themes	Subthemes
Engagement & initial perceptions	Excitement & curiosity about D&D	Staff curiosity; novelty of approach; relevance to pupils already interested in D&D
	Positive reception & buy-in	Early enthusiasm from pupils; rapid engagement; willingness to try
	Parental perceptions & consent	Face-to-face & email communication; initial scepticism vs. later support
Social interaction & peer relationships	Intra-group dynamics	Bonding across classes; teasing & camaraderie; handling interpersonal conflict
	Collaboration & teamwork	Turn-taking; listening skills; problem-solving together
	Cross-cultural exchange	Learning from culturally diverse peers; inclusive participation
Personal development & behavioural change	Emotional regulation	Calmer behaviour; self-control; fewer behavioural incidents
	Self-management	Improved lesson readiness; reduced exclusions; better instruction-following
	Confidence & self-expression	Increased social participation; role-play enabling safe expression
	Perspective shifts	Changes in perception of authority figures; feeling understood
Facilitator & programme design factors	Skilled facilitation	Enthusiastic, informal, and structured leadership
	Structure & pacing	Breaking tasks into steps; clear expectations; safe environment
	Optimal conditions	Appropriate age groups; session length; number of participants
Outcomes, sustainability & unintended effects	Positive spillover	Improved relationships outside the game; parental observations of home behaviour
	Unintended outcomes	Strengthened adult–student rapport; peer fallouts unrelated to programme
	Sustainability & scalability	Need for longer interventions; expansion to more pupils; integration into wider wellbeing strategies

Teacher and facilitator accounts converged on strong engagement, small group safety and gains in self-management, teamwork, confidence and social connection for many participants, with variability linked to facilitation, group composition and scheduling. Staff described shifts from competition to collaboration (“*‘I want to go first’… now they understand they have to take turns*,* they have to work as a team”*), improved emotional regulation (“being able to stop, understand and follow instructions”) and increased confidence and voice among previously quiet pupils (“*he’s very vocal… speaks with a louder voice and his head held up high”).* A parent who was also a member of staff in a participating school noted spillover beyond sessions (“*he was quite calm… he listened and engaged”; “even at home he was more compliant”).* The D&D format was viewed as therapeutic without stigma, combining structure and imaginative freedom in a psychologically safe space, though early hesitancy, selection stigma and timetable/resource constraints were reported. Facilitator skill was repeatedly identified as critical to holding boundaries, pacing narratives and adapting challenges to developmental level.

Mapping of interview material to CASEL competencies indicated the most salient domains were self-management (eight instances) and relationship skills (seven), followed by responsible decision making (four), with fewer explicit references to self-awareness and social awareness (two each). This profile mirrors the quantitative signals in anger control, peer support and cooperation. Stakeholders expressed a desire to continue and expand provision, paired with recognition that sustainability will require predictable scheduling, dedicated space and capacity building for trained delivery.

### Mixed methods integration

The integrated joint displays aligned three recurrent quantitative and qualitative patterns. First, improved anger control and cooperation for a substantial subgroup cooccurred with teacher observed coregulation and turn taking in small, stable groups led by skilled facilitators. Second, stable or strengthened belonging and enjoyment for many pupils coexisted with a minority shift toward low categories. Although individual-level linkage was not directly assessed, it was hypothesised from the interviews to that contextual factors including divergence to timetable pressure, unfamiliar adults, and space changes may have blunted engagement. Third, modest gains in competence self-appraisal and “good qualities” mapped to vignettes of confidence and leadership emergence, especially among previously withdrawn pupils, while the small rise in strong negative self-appraisals was interpreted as increased self-reflection for a few. These patterns support provisional context–mechanism–outcome propositions to be tested in a longer follow up: small, consistent groups and skilled facilitation (context) trigger coregulated play and perspective taking (mechanism), producing improved self-management and teamwork (outcome); delivery instability (context) dampens the same mechanisms, attenuating belonging and engagement (outcome).

A systematic review of the transcripts identified 8 instances of self-management, 7 of relationship skills, 4 of responsible decision-making, 2 of social awareness and 2 of self-awareness. These counts represent explicit, observable behaviours or reflections aligned with each CASEL competency and provide insight into the areas where the programme’s impact was most evident. Table 6 presents a representative quote from each CASEL domain including the relative frequency of each competency observed in the dataset.

**Table 6:** Quote matrix and frequency distribution (CASEL framework)

Competency	f	Theme	Quote	Source
Self-awareness	2	Recognizing emotions and strengths	One student said that they believed that “my self-management has improved, and I can see it…” What they lacked was being able to regulate their emotions, being able to stop, understand and follow instructions.	Westminster Academy
Self-awareness		Reflecting on self through role-play	They said, “It was the highlight of my week…” It was an opportunity for them to express themselves in a safe environment through a character… and learn how that impacts other people.	Learning support assistant ‚ St Marylebone
Self-management	8	Improved emotional regulation	In the very beginning they would argue‚ but now they understand they have to take turns, they have to work as a team using their confidence in wanting to work together rather than against each other.	Westminster Academy
		Transfer of regulation skills to home	Even at home‚ they are more compliant and better at understanding instructions.	Parent feedback via Westminster Academy
Social awareness	2	Empathy and cross-group interaction	He was more social in that group… he spoke to children from the other class. He was excited about it.	Parent at St Francis
		Appreciating different perspectives	It captured their imagination‚. It allowed expression and imagination to flourish.	Learning support assistant, St Marylebone
Relationship skills	7	Improved peer-staff relationships	One student didn’t really speak to me at all, now she waves, engages in conversation, is friendly.	Learning support assistant, St Marylebone
		Positive facilitation and group cohesion	Rupert was very good. He was skilled at being friends with the girls, holding the game and making the experience enjoyable for everybody.	Learning support assistant Marylebone
Responsible decision-making	4	Making constructive choices	Sometimes they might use that confidence to say what’s on their mind happen, allowing them to step into the right places.	Westminster Academy
		Collaborative problem-solving	They had to figure out how to get back from the island. Him and his friend done it together.	Parent at St Francis

## Discussion

### Principal findings

This convergent realist mixed-methods pilot evaluated the Young Dragons tabletop role-playing intervention for pupils at risk of exclusion across ten state schools in WCC and RBKC. Quantitative results did not show any statistically significant average pre-post changes across matched pairs, yet distributions showed simultaneous improvement and deterioration in emotional wellbeing, self-concept, anger control and school belonging. In addition to pupil-reported outcomes, routine school records provided a complementary behavioural signal. Across 30 matched pupils participating in the intervention with available attendance and suspension data, mean attendance increased from 90.6% to 92.7% over the intervention period, although this change did not reach conventional statistical significance (*p* = 0.069). More notably, mean suspensions declined significantly from 0.7 pre-intervention to 0.0 post-intervention (*p* = 0.022). While these findings should be interpreted cautiously given the small sample and short follow-up, they are directionally consistent with staff accounts describing calmer behaviour, improved emotional regulation and fewer behavioural escalations during and after sessions. Within a realist interpretation, these patterns suggest that the programme may exert indirect effects on behavioural stability through mechanisms of co-regulation, turn-taking and increased psychological safety, even over a brief delivery window. This quantitative signal from observations relating primarily to participating pupils, with occasional reference to broader classroom dynamics, aligns with school narratives describing calmer behaviour, improved regulation and fewer behavioural escalations.

Qualitative accounts described strong engagement, psychological safety and visible gains in self-management, teamwork and peer connection for many participants, alongside implementation challenges related to scheduling, space and facilitator stability. Integration of both strands supports a mechanism-based interpretation- that outcomes were conditioned by context and facilitation rather than uniform programme effects. Collectively, these findings strengthen the case that Young Dragons may indirectly support attendance and behavioural stability even within a short timeframe.

### Interpretation of quantitative and qualitative results

The absence of statistically significant mean change reflects the design and sample rather than lack of impact. With only 22 matched pairs and a six-to-eight-week exposure window, the analysis was under-powered for conventional significance testing. However, the directionality of change and category shifts point to a phenomenon of polarisation, typical in developmental and psychosocial pilots where some pupils thrive while others show transient discomfort or disengagement.

Teachers and facilitators described the same heterogeneity: pupils who entered sessions withdrawn often became more expressive and cooperative, while a smaller subset appeared unsettled by introspection or the unstructured nature of imaginative play. The programme’s mechanisms of narrative immersion, cooperative rule-based interaction and structured reflection appear to benefit pupils ready for social learning, while those with unresolved distress or unstable attendance may require longer exposure and additional containment to avoid reactive stress. This nuanced picture illustrates why early-stage, creative interventions should be interpreted through a realist rather than positivist lens.

### Mechanisms of change

Findings align closely with the intervention’s theory of change and with SEL scholarship. The Young Dragons format created a predictable, rule-governed micro-community that exercised the CASEL competencies: (i) Self-management emerged through anger control, turn-taking and behavioural regulation, (ii) Relationship skills through collaboration and empathy in shared quests, (iii) Self-awareness through character creation and “safe self-projection”, and (iv) Responsible decision-making through simulated moral and strategic dilemmas.

Teachers linked positive behavioural changes to these mechanisms, noting calmer classroom conduct and renewed confidence among participants. Where the setting was disrupted by room changes, timetable clashes, or untrained facilitators the same mechanisms were blunted and pupils’ sense of belonging declined. In realist terms, the context (stable group, skilled facilitator, consistent space) triggered mechanisms of co-regulation and perspective-taking that yielded positive outcomes, while adverse contexts interrupted these chains.

### Comparison with existing evidence

The findings corroborate international literature demonstrating TTRPGs can enhance social confidence, empathy and teamwork through narrative collaboration [21]. Studies in therapeutic and community settings have reported reduced anxiety and improved self-esteem after Dungeons & Dragons interventions [17, 21]. Similar school-based experiments in Australia and North America show increases in communication skills and prosocial behaviour [23–25]. The present study extends this evidence into the UK state-school context and adds teachers’ perspectives, which are scarce but essential for implementation science [19].

Unlike earlier research in after-school or clinical environments, this pilot occurred during standard timetable hours, within high-need urban schools characterised by linguistic diversity and resource constraints. That the intervention was both feasible and welcomed by staff indicates that TTRPG-based SEL programmes can translate beyond voluntary clubs into mainstream inclusion policy, provided their delivery is carefully scaffolded.

#### Influence of local context

WCC and RBKC present unique conditions for evaluating creative, low-stigma mental-health interventions. Despite their overall affluence, both boroughs exhibit high deprivation pockets, elevated free-school-meal eligibility and one of the highest proportions of pupils with English as an additional language in England. Demand for child mental-health services far exceeds capacity. In this environment, imaginative group interventions can offer accessible early support that complements overstretched specialist services.

The pilot’s success in operating across ten schools demonstrates adaptability to these complex systems. However, logistical friction competing timetable priorities, room availability and fluctuating staff support significantly influenced outcomes. Such contextual realities reinforce that scalability depends not only on curriculum fit but also on administrative and infrastructural readiness.

### Implementation lessons

The evaluation identifies several actionable levers for practitioners and commissioners. In terms of facilitation quality, teacher narratives converge on the importance of highly skilled facilitators able to manage behaviour, maintain narrative coherence and balance therapeutic empathy with classroom discipline [26]. Group size and consistency were also important as **c**ohorts of four to six pupils were optimal for engagement and safety. Larger or unstable groups risked dominance by a few voices and loss of trust. Environmental stability is deemed critical as consistent time slots and dedicated rooms create predictability essential for pupils with SEMH needs [27, 28]. Stigma-free recruitment was essential, including **l**abelling sessions as “creative storytelling” or “leadership” rather than “intervention” improved acceptability among families and pupils. Finally, bridging to the classroom using structured reflection linking in-game behaviour to real-life contexts could strengthen skill transfer and allow teachers to reinforce lessons learned [26, 28]. These levers correspond to known fidelity drivers in SEL implementation research and provide a roadmap for integrating TTRPG-based learning within inclusive-education strategies [28].

#### Strengths and limitations of the study

The study’s strengths lie in its ecological validity and methodological integration. It was embedded in routine school practice, spanned multiple educational phases and engaged high-need populations under real delivery conditions. The convergent mixed-methods design allowed quantitative heterogeneity to be interpreted through qualitative mechanism analysis, preventing premature conclusions from under-powered statistical testing. The realist orientation generated explanatory hypotheses rather than binary verdicts and triangulation between teacher interviews, facilitator reflections and steering-group observations increased trustworthiness [29]. Finally, the study demonstrated that brief, child-friendly surveys can be administered feasibly in classroom settings to capture SEL-relevant constructs.

The principal limitation of this study is the single-arm pre–post structure precludes causal inference; regression to the mean and concurrent school factors cannot be ruled out. The small matched-pair sample and attrition between timepoints reduce statistical power. Survey responses may reflect transient mood or social desirability, especially in multilingual cohorts with varying literacy. Ordinal scales compress variance and may not capture nuanced change. Exposure of only six to eight weeks may be insufficient for sustained social-emotional development and long-term outcomes remain unknown [8]. The study also did not assess baseline familiarity with or interest in tabletop role-playing games, which may have influenced engagement and response trajectories. We acknowledge also that the absence of follow-up measurement limits assessment of durability of effects and whether observed changes persist, attenuate, or consolidate over time.

In the qualitative component, the necessity to avoid intrusive observation reduced opportunities for behavioural coding and inclusion of two facilitators among interviewees may have introduced positive bias [30]. Nevertheless, reflexive documentation and triangulation mitigated these risks. Qualitative findings were derived from a small sample (*n* = 9), including only three teachers, and may reflect role-specific perspectives. Observations were also not consistently linked to individual pupils, limiting attribution of reported changes to specific participants and raising the possibility of broader classroom-level spillover effects. Overall, the evaluation should be viewed as a feasibility and mechanism study rather than a definitive test of effectiveness.

#### Implications for theory

The study adds to the theoretical understanding of how narrative play facilitates social-emotional learning. It supports models of co-regulated learning where structured fantasy environments allow pupils to practise emotional regulation and perspective-taking without real-world penalty. The observation of “polarisation” parallels developmental models in which increased self-reflection temporarily heightens distress before consolidation, an effect reported in expressive and drama-based therapies. The realist CMO mapping derived here can inform refinement of programme theory: (i) Context - small, stable groups; skilled facilitation; consistent environment, (ii) Mechanism - co-regulated play, narrative identity exploration, perspective-taking and (iii) Outcome - improved self-management, confidence, teamwork and belonging. Conversely, when contextual supports weaken, mechanisms misfire, producing disengagement or transient distress. These propositions generate empirically testable hypotheses for the next stage of research.

#### Policy and practice implications

At policy level, Young Dragons aligns with the UK Government’s inclusive-education and mental-health-in-schools agendas. It operationalises prevention within the school day and demonstrates how core public-health principles - early intervention, low stigma and co-production - can be translated into practice through creative pedagogy. Commissioners seeking to reduce exclusions should consider TTRPG-based interventions as part of multi-tiered support systems, ensuring that procurement specifies facilitator qualifications, safeguarding standards and fidelity monitoring.

For schools, embedding requires structural support: protected time within the timetable, leadership buy-in and trained internal champions. Teachers involved in this study viewed the programme as complementing existing pastoral initiatives rather than competing with curriculum time. For sustainability, a train-the-trainer model could develop internal capacity while maintaining quality assurance through external supervision. Data from this evaluation can guide such scaling by identifying feasible group sizes, session length and materials adaptable to different literacy and language levels.

### Future research directions

A rigorous next-phase study should employ a quasi-experimental or stepped-wedge design across multiple schools to test the causal validity of observed mechanisms. Outcome measurement should broaden to include teacher-rated behavioural scales, attendance records and disciplinary data, alongside pupil self-reports. Fidelity monitoring, facilitator competence checklists and contextual audits will allow multilevel modelling of variation. Longitudinal follow-up over at least one academic term is needed to detect “sleeper” effects and potential fade-out, as documented in broader SEL literature. Given that the way that pupils are supported through self-discovery and introspection is critical so that it remains psychologically safe is important, future studies could look at whether a universal school approach is more inclusive. Mixed-methods integration should continue, aligning quantitative trajectories with qualitative narratives through joint displays to refine CMO configurations, whilst also looking to investigate cultural appropriateness of the D&D in different groups. Finally, cost-effectiveness and equity analyses are essential to ensure scalability within resource-constrained local authorities.

## Conclusion

Young Dragons demonstrates that tabletop role-playing (TTRPG), narrative-based interventions can be delivered feasibly and acceptably within UK state schools serving pupils at risk of exclusion. Although statistically significant group-level changes in self-reported wellbeing were not detected, mixed-methods integration revealed mechanism-consistent improvements for many participants, particularly in self-management, cooperation, confidence and peer relationships, when delivery conditions were supportive. Outcomes were contingent on context, including facilitator skill, group stability and environmental predictability, underscoring the importance of implementation quality in creative, school-based mental health interventions.

Consistent with staff narratives of calmer behaviour and fewer behavioural escalations, exploratory analysis of routine school data showed a significant reduction in suspensions and a modest, non-significant increase in attendance over the intervention period, suggesting potential indirect effects on behavioural stability even within a short timeframe. While these findings should be interpreted cautiously, they strengthen the plausibility that narrative, co-regulated play may influence downstream educational outcomes through improved emotional regulation and group functioning.

Taken together, this pilot provides a robust feasibility and mechanism-mapping foundation for a next-phase evaluation. Future studies should employ controlled or stepped-wedge designs, incorporate longer follow-up and link pupil-reported outcomes to administrative indicators such as attendance, behaviour and exclusion. With appropriate investment in facilitator training and delivery stability, TTRPG-based interventions such as Young Dragons may form a scalable component of inclusive, preventive mental-health strategies within contemporary school systems.

## Supplementary Information


Supplementary Material 1.



Supplementary Material 2.



Supplementary Material 3.



Supplementary Material 4.


## Data Availability

The datasets generated and/or analysed during the current study are not publicly available due to ethical and governance restrictions. The study involves minors in school settings, and the quantitative survey data, qualitative interview transcripts, and routine school records contain information that could potentially enable participant or school identification if shared openly. Data access is therefore restricted under the terms of approval granted by the Imperial College Research Ethics Committee and data-sharing agreements with participating schools and local authorities. De-identified and aggregated data supporting the findings of this study may be made available from the corresponding author on reasonable request, subject to review of the request, approval by the relevant data controllers, and completion of appropriate data-sharing agreements. The survey instrument used in the study is provided as **Supplementary File 1.**.

## Abbreviations

ARC: Applied Research Collaboration
CASEL: Collaborative for Academic, Social, and Emotional Learning
CMO: Context–Mechanism–Outcome
COREQ: Consolidated Criteria for Reporting Qualitative Research
D&D: Dungeons & Dragons
EHCP: Education, Health and Care Plan
EAL: English as an Additional Language
FSM: Free School Meals
GRAMMS: Good Reporting of A Mixed Methods Study
ICREC: Imperial College Research Ethics Committee
LSA: Learning Support Assistant
NIHR: National Institute for Health and Care Research
PRU: Pupil Referral Unit
RBKC: Royal Borough of Kensington and Chelsea
SEL: Social and Emotional Learning
SEND: Special Educational Needs and Disabilities
SEMH: Social, Emotional and Mental Health
STROBE: Strengthening the Reporting of Observational Studies in Epidemiology
TIDieR: Template for Intervention Description and Replication
TTRPG: Tabletop Role–Playing Game
WCC: Westminster City Council
