# Supplementary material for: Imaginative play for inclusion: evaluating the young dragons tabletop role-playing intervention in UK schools
Source: BMC Public Health. 2026 Jun 20;26:2497. doi: 10.1186/s12889-026-28225-z (PMC13531801; doi:10.1186/s12889-026-28225-z)
Supplement: Supplementary file 1 [file 12889_2026_28225_MOESM2_ESM.pdf]

Supplementary File 2: STROBE Checklist

Strengthening the Reporting of Observational Studies in Epidemiology

Title of manuscript: Imaginative play for inclusion: Evaluating the Young Dragons tabletop role-playing intervention in UK schools

Study design: Single-arm pre–post observational pilot study (mixed-methods)

Item No.	Recommendation	Page No(s).	Location / Description
1(a)	Indicate the study's design in the title or abstract	1	Abstract – Methods ("convergent mixed-methods realist evaluation; pre–post")
1(b)	Provide an informative and balanced abstract	1	Structured abstract
2	Explain the scientific background and rationale	2–4	Introduction
3	State specific objectives	4	Introduction – final paragraph
4	Present key elements of study design early	5	Methods – Study design
5	Describe setting, locations, and dates	5–6	Methods – Setting, participants and recruitment
6(a)	Give eligibility criteria and selection methods	6	Methods – Setting, participants and recruitment
6(b)	Matching criteria (if applicable)	NA	Not applicable (no comparison group)
7	Clearly define all outcomes and variables	7	Methods – Quantitative measures
8	Data sources and measurement methods	7–8	Methods – Quantitative measures and data collection
9	Describe efforts to address bias	5, 18	Methods – Study design; Discussion – Limitations
10	Explain how study size was arrived at	5	Methods – Study design (pilot feasibility)
11	Explain handling of quantitative variables	8	Methods – Quantitative analysis
12(a)	Describe statistical methods	8	Methods – Quantitative analysis
12(b)	Methods for subgroup analyses	NA	Not conducted
12(c)	Explain handling of missing data	7, 9	Methods – Data collection; Results – Participants

12(d)	Address loss to follow-up	9	Results – Participants and data completeness
12(e)	Sensitivity analyses	NA	Not conducted
13(a)	Report numbers at each stage	9	Results – Participants
13(b)	Reasons for non-participation	9	Results – Participants
13(c)	Flow diagram	NA	Not included (pilot study)
14(a)	Participant characteristics	9–10	Results; Table 3
14(b)	Indicate missing data	9	Results – Participants
15	Report outcome data	10–12	Results – Quantitative outcomes
16(a)	Give unadjusted estimates and precision	11–13	Results – Tables 1–2
16(b)	Category boundaries	7	Methods – Quantitative measures
16(c)	Relative risk translation	NA	Not applicable
17	Report other analyses	12	Results – Responder heterogeneity
18	Summarise key results	14–15	Discussion – Principal findings
19	Discuss limitations	18	Discussion – Strengths and limitations
20	Interpretation in context	14–18	Discussion
21	Discuss generalisability	18–19	Discussion – Policy and practice implications
22	Funding	21	Declarations – Funding
