# Supplementary material for: Imaginative play for inclusion: evaluating the young dragons tabletop role-playing intervention in UK schools
Source: BMC Public Health. 2026 Jun 20;26:2497. doi: 10.1186/s12889-026-28225-z (PMC13531801; doi:10.1186/s12889-026-28225-z)
Supplement: Supplementary file 2 [file 12889_2026_28225_MOESM3_ESM.pdf]

Supplementary File 3: COREQ Checklist

Consolidated Criteria for Reporting Qualitative Research
Approach: Semi-structured interviews and non-participant observation
Analysis: Thematic analysis (Braun & Clarke)

Item	Description	Page No(s).	Location / Description
1	Interviewer/facilitator	8	Methods – Qualitative design
2	Credentials	1	Title page
3	Occupation	1	Author affiliations
4	Gender	NA	Not reported
5	Experience/training	8	Methods – Qualitative design
6	Relationship with participants	8	Methods – Qualitative design
7	Participant knowledge of interviewer	8	Methods – Qualitative design
8	Interviewer characteristics	NA	Not explicitly described
9	Methodological orientation	8	Methods – Qualitative design
10	Sampling	8	Methods – Qualitative design
11	Method of approach	8	Methods – Qualitative design
12	Sample size	13	Results – Qualitative outcomes
13	Non-participation	NA	Not applicable
14	Setting	8	Methods – Qualitative design
15	Presence of non-participants	8	Methods – Qualitative design
16	Description of sample	13	Table 4
17	Interview guide	8	Methods – Qualitative design
18	Repeat interviews	NA	Not conducted
19	Audio/visual recording	8	Methods – Qualitative design
20	Field notes	8	Methods – Qualitative design
21	Duration	8	Methods – Qualitative design
22	Data saturation	9	Methods – Qualitative analysis
23	Transcripts returned	NA	Not conducted

24	Number of data coders	9	Methods – Qualitative analysis
25	Coding tree	NA	Not presented
26	Derivation of themes	9	Methods – Qualitative analysis
27	Software	9	Methods – Qualitative analysis
28	Participant checking	NA	Not conducted
29	Quotations presented	13–16	Results – Qualitative outcomes
30	Consistency between data and findings	13–16	Results – Qualitative outcomes
31	Clarity of major themes	14	Table 5
32	Clarity of minor themes	15	Table 6
