# Supplementary material for: Imaginative play for inclusion: evaluating the young dragons tabletop role-playing intervention in UK schools
Source: BMC Public Health. 2026 Jun 20;26:2497. doi: 10.1186/s12889-026-28225-z (PMC13531801; doi:10.1186/s12889-026-28225-z)
Supplement: Supplementary file 3 [file 12889_2026_28225_MOESM4_ESM.pdf]

Post_Young Dragons Pupils

Start of Block: Default Question Block

Q1 This survey helps us understand how you are feeling, how things are going at school, and what you think about the Young Dragons sessions. There are no right or wrong answers. Just answer as honestly as you can. If you are not sure, that's okay too 😌 Post_I_Survey

---

Q2 Would you like to complete this simple survey? It's ok if you don't want to.

○ Yes (1)

○ No (2)

Skip To: End of Survey If Would you like to complete this simple survey? It's ok if you don't want to. = No

---

Q3 What is your Hero name?

____

____

____

____

____

End of Block: Default Question Block

Start of Block: Block 8

Q4 Please help us understand how you have been feeling in the last week

	☹ 👎👎 (1)	👎 (3)	😐 (6)	👍 (8)	😊 👍👍 (9)
How is everything going? (4)	○	○	○	○	○
How are you doing at school? (3)	○	○	○	○	○
How are things in your family? (2)	○	○	○	○	○
How are you doing? (1)	○	○	○	○	○

End of Block: Block 8

Start of Block: Bee-Well

Q5 Please tell us how you think and feel about yourself.

	Strongly agree 👍👍 (1)	Agree 👍 (2)	😐 I'm usnure (3)	Disagree 👎 (4)	Strongly disagree 👎👎 (5)
I am satisfied with myself (1)	○	○	○	○	○
I have a number of good qualities (2)	○	○	○	○	○
I am able to do things as well as most other people (3)	○	○	○	○	○
I feel good about myself (5)	○	○	○	○	○

Page Break

Q6 Remember if you do not want to answer a question, you can skip it. How true are the following statements?

	Very not true 👎👎 (1)	Not true 👎 (2)	😐 I'm unsure (3)	True 👍 (5)	Very True 👍👍 (6)
I find it hard to control my feelings (1)	○	○	○	○	○
I'm able to deal with stress (3)	○	○	○	○	○
I can control my anger when I want to (4)	○	○	○	○	○

Page Break

Q7 How would you answer the following statements?

	Not at all 👎 (1)	A little 👍 (2)	Quite a bit 👍👍 (4)	A lot 👍👍👍 (5)
I get along with people around me (1)	○	○	○	○
People like to spend time with me (2)	○	○	○	○
I feel supported by my friends (3)	○	○	○	○
My friends care about me when times are hard (4)	○	○	○	○
I take turns when playing games with others (5)	○	○	○	○

End of Block: Bee-Well

Start of Block: Social connection

Q8 How often do you feel lonely?

○ Never (1)

○ Rarely (2)

○ Occasionally (3)

○ Some of the time (4)

○ Often or always (5)

End of Block: Social connection

Start of Block: School engagement

Q9 I feel like I belong at my school 💖

◯ Not at all true (1)

◯ A little true (2)

◯ Mostly true (3)

◯ Completely true (4)

◯ I'm not sure (5)

Q10 I enjoy coming to school 🏫

◯ Not at all (1)

◯ A little (2)

◯ Sometimes (3)

◯ A lot (4)

◯ I'm not sure (5)

Q11 Well done for completing the survey. You can raise your hand up if you like, so we know you are finished. 😊

End of Block: School engagement
