# Supplementary material for: Imaginative play for inclusion: evaluating the young dragons tabletop role-playing intervention in UK schools
Source: BMC Public Health. 2026 Jun 20;26:2497. doi: 10.1186/s12889-026-28225-z (PMC13531801; doi:10.1186/s12889-026-28225-z)
Supplement: Supplementary file 4 [file 12889_2026_28225_MOESM1_ESM.docx]

**Supplementary Table 1: Main survey findings (n=22 matched participants)**

| **Variable** | **Frequency (percentage)** | |
| --- | --- | --- |
|  | **Before** | **After** |
| **Please help us understand how you have been feeling in the last week** | | |
| **- How is everything going?** | | |
| ☹️ 👎👎 | 0 (0.0%) | 2 (9.1%) |
| 👎 | 1 (4.5%) | 0 (0.0%) |
| 😐 | 1 (4.5%) | 2 (9.1%) |
| 👍 | 11 (50.0%) | 6 (27.3%) |
| 😊 👍👍 | 9 (40.9%) | 12 (54.5%) |
| **- How are you doing at school?** | | |
| ☹️ 👎👎 | 0 (0.0%) | 3 (13.6%) |
| 👎 | 0 (0.0%) | 1 (4.5%) |
| 😐 | 6 (27.3%) | 3 (13.6%) |
| 👍 | 10 (45.5%) | 8 (36.4%) |
| 😊 👍👍 | 6 (27.3%) | 7 (31.8%) |
| **- How are things in your family?** | | |
| ☹️ 👎👎 | 1 (4.5%) | 0 (0.0%) |
| 👎 | 0 (0.0%) | 0 (0.0%) |
| 😐 | 3 (13.6%) | 0 (0.0%) |
| 👍 | 3 (13.6%) | 8 (36.4%) |
| 😊 👍👍 | 15 (68.2%) | 14 (63.6%) |
| **- How are you doing?** | | |
| ☹️ 👎👎 | 0 (0.0%) | 1 (4.5%) |
| 👎 | 1 (4.5%) | 0 (0.0%) |
| 😐 | 5 (22.7%) | 3 (13.6%) |
| 👍 | 10 (45.5%) | 7 (31.8%) |
| 😊 👍👍 | 6 (27.3%) | 11 (50.0%) |
| **Please tell us how you think and feel about yourself.** | | |
| **- I am satisfied with myself** | | |
| Strongly agree | 7 (31.8%) | 0 (0.0%) |
| Agree | 9 (40.9%) | 9 (40.9%) |
| I'm unsure | 5 (22.7%) | 4 (18.2%) |
| Disagree |  |  |
| Strongly disagree | 1 (4.5%) | 1 (4.5%) |
| **- I have a number of good qualities** | | |
| Strongly agree | 3 (13.6%) | 4 (18.2%) |
| Agree | 11 (50.0%) | 14 (63.6%) |
| I'm unsure | 7 (31.8%) | 3 (13.6%) |
| Disagree | 0 (0.0%) | 1 (4.5%) |
| Strongly disagree | 1 (4.5%) | 0 (0.0%) |
| **- I am able to do things as well as most other people** | | |
| Strongly agree | 5 (22.7%) | 5 (22.7%) |
| Agree | 6 (27.3%) | 11 (50.0.0%) |
| I'm unsure | 5 (22.7%) | 4 (18.2%) |
| Disagree | 3 (13.6%) | 2 (9.1%) |
| Strongly disagree | 3 (13.6%) | 0 (0.0%) |
| **- I feel good about myself** |  |  |
| Strongly agree | 8 (36.4%) | 8 (36.4%) |
| Agree | 10 (45.5%) | 9 (40.9%) |
| I'm unsure | 2 (9.1%) | 3 (13.6%) |
| Disagree | 1 (4.5%) | 1 (4.5%) |
| Strongly disagree | 1 (4.5%) | 1 (4.5%) |
| **Remember if you do not want to answer a question, you can skip it. How true are the following statements?** | | |
| **- I find it hard to control my feelings** | | |
| Very not true | 2 (9.1%) | 2 (9.1%) |
| Not true | 4 (18.2%) | 6 (27.3%) |
| I'm unsure | 11 (50.0%) | 8 (36.4%) |
| True | 2 (9.1%) | 4 (18.2%) |
| Very True | 3 (13.6%) | 2 (9.1%) |
| **- I'm able to deal with stress** | | |
| Very not true | 3 (13.6%) | 1 (4.5%) |
| Not true | 5 (22.7%) | 5 (22.7%) |
| I'm unsure | 5 (22.7%) | 10 (45.5%) |
| True | 8 (36.4%) | 4 (18.2%) |
| Very True | 1 (4.5%) | 2 (9.1%) |
| **- I can control my anger when I want to** | | |
| Very not true | 3 (13.6%) | 2 (9.1%) |
| Not true | 3 (13.6%) | 1 (4.5%) |
| I'm unsure | 5 (22.7%) | 8 (36.4%) |
| True | 5 (22.7%) | 7 (31.8%) |
| Very True | 6 (27.3%) | 4 (18.2%) |
| **How would you answer the following statements?** | | |
| **- I get along with people around me** | | |
| Not at all | 0 (0.0%) | 0 (0.0%) |
| A little | 7 (31.8%) | 3 (13.6%) |
| Quite a bit | 5 (22.7%) | 9 (40.9%) |
| A lot | 10 (45.5%) | 10 (45.5%) |
| **- People like to spend time with me** | | |
| Not at all | 0 (0.0%) | 1 (4.5%) |
| A little | 8 (36.4%) | 3 (13.6%) |
| Quite a bit | 6 (27.3%) | 8 (36.4%) |
| A lot | 8 (36.4%) | 10 (45.5%) |
| **- I feel supported by my friends** | | |
| Not at all | 1 (4.5%) | 1 (4.5%) |
| A little | 5 (22.7%) | 4 (18.2%) |
| Quite a bit | 5 (22.7%) | 4 (18.2%) |
| A lot | 11 (50.0.0%) | 13 (59.1%) |
| **- My friends care about me when times are hard** | | |
| Not at all | 1 (4.5%) | 2 (9.1%) |
| A little | 4 (18.2%) | 3 (13.6%) |
| Quite a bit | 7 (31.8%) | 5 (22.7%) |
| A lot | 10 (45.5%) | 12 (54.5%) |
| **- I take turns when playing games with others** | | |
| Not at all | 1 (4.5%) | 1 (4.5%) |
| A little | 5 (22.7%) | 4 (18.2%) |
| Quite a bit | 6 (27.3%) | 6 (27.3%) |
| A lot | 10 (45.5%) | 11 (50.0%) |
| **How often do you feel lonely?** | | |
| Never | 3 (13.6%) | 5 (22.7%) |
| Rarely | 9 (40.9%) | 8 (36.4%) |
| Occasionally | 5 (22.7%) | 6 (27.3%) |
| Some of the time | 5 (22.7%) | 2 (9.1%) |
| Often or always | 0 (0.0%) | 1 (4.5%) |
| **I feel like I belong at my school** | | |
| N-Miss | 1 | 0 |
| Not at all true | 3 (14.3%) | 3 (13.6%) |
| A little true | 4 (19.0%) | 2 (9.1%) |
| Mostly true | 7 (33.3%) | 8 (36.4%) |
| Completely true | 7 (33.3%) | 9 (40.9%) |
| I'm not sure | 0 (0.0%) | 0 (0.0%) |
| **I enjoy coming to school** | | |
| N-Miss | 1 | 0 |
| Not at all | 6 (28.6%) | 4 (18.2%) |
| A little | 1 (4.8%) | 1 (4.5%) |
| Sometimes | 6 (28.6%) | 9 (40.9%) |
| A lot | 6 (28.6%) | 8 (36.4%) |
| I'm not sure | 2 (9.5%) | 0 (0.0%) |
